# Relapsing polychondritis patients were divided into three subgroups: patients with respiratory involvement (R subgroup), patients with auricular involvement (A subgroup), and overlapping patients with both involvements (O subgroup), and each group had distinctive clinical characteristics

**DOI:** 10.1097/MD.0000000000012837

**Published:** 2018-10-19

**Authors:** Jun Shimizu, Yoshihisa Yamano, Kimito Kawahata, Noboru Suzuki

**Affiliations:** Department of Immunology and Medicine, Institute of Medical Science, and Division of Rheumatology and Allergology, St. Marianna University School of Medicine, Kawasaki, Japan.

**Keywords:** auricular involvement, cardiovascular involvement, inflammation, relapsing polychondritis, respiratory involvement

## Abstract

Relapsing polychondritis (RP) is a multisystem disorder of cartilaginous tissues. Previously, we found that patients with respiratory involvement and patients with auricular involvement were mutually exclusive in the RP cohort, which suggests a strong inverse relationship between respiratory and auricular involvement. Here, we examined the clinical manifestation patterns in a subgroup of patients with respiratory involvement (R subgroup) and a subgroup of patients with auricular involvement (A subgroup) and investigated the clinical and laboratory characteristics of each subgroup.

There were 47 patients (19.7%) and 118 patients (49.4%) allocated to the R and A subgroups, respectively. Saddle nose deformity and a progressive disease course were observed frequently in the R subgroup. Arthritis, conjunctivitis, and CNS involvement were observed frequently in the A subgroup.

The remaining RP patients formed a third subgroup of patients that had both respiratory involvement and auricular involvement. We designated this subgroup as the O (overlap) subgroup, and 75 patients (31.4%) were allocated to the O subgroup. Disease duration in the O subgroup (5.70 ± 0.64 years) was significantly longer than that in the A subgroup (4.12 ± 0.45 years) and relatively longer than that in the R subgroup (4.80 ± 0.63 years).

We found that cardiovascular involvement was more predominant in the O subgroup than in the R and A subgroups. Higher concentrations of serum matrix metalloproteinase (MMP)3 were observed in the O subgroup than in the R and A subgroups.

We measured serum MMP3 concentrations in another patient cohort including 22 newly recruited RP patients. MMP3 concentrations were significantly higher in the O subgroup (n = 10) than those in the R subgroup (n = 6) and A subgroup (n = 10).

RP patients in the R and A subgroups had different characteristics from each other, and the overlap of respiratory and auricular involvement was an important prognostic factor in patients with RP. Cardiovascular involvement was not observed in the R subgroup in RP patients. The current study may provide insights into the classification and treatment of RP.

## 1 Introduction

Relapsing polychondritis (RP) is a multisystem disorder characterized by recurrent inflammation and degeneration of cartilaginous tissues such as the ear, nose, joint, and respiratory tract.^[1]^ RP may affect other proteoglycan rich organs such as the eye, inner ear, heart, blood vessels, and kidney.^[1]^

We conducted an epidemiological survey of 239 RP patients and collected clinical information.^[2]^ We reported that respiratory failure with pulmonary infection was a major cause of death in patients with RP.^[2]^ Although the incidence was lower than that in a Caucasian study,^[3]^ central nervous system (CNS) involvement,^[4]^ and cardiovascular system involvement^[5]^ were important prognostic factors in Japan. Cardiovascular involvement frequently occurred with CNS, auricular, and kidney involvement.^[5]^

Francès et al^[6]^ reported that a relatively large number of French RP patients (36.5%) had chronic dermatitis, and these patients suffered frequently from hematological disorders (12.0%), especially myelodysplastic syndrome (MDS, 11.0%) and connective tissue diseases (11.0%).

Our previous study showed that 32 RP patients (13.8%) developed cutaneous manifestations and 5 patients (2.1%) had MDS.^[7]^ All 5 patients with MDS had cutaneous manifestations.

Recently, the same French group reported that, using a cluster analysis, 142 RP patents were characterized by three different clinical phenotypes. Of the three phenotypes, the hematological cluster, which had a large number of patients with MDS, had the poorest outcomes.^[8]^

We examined how organ involvement in RP patients was associated with one another. We assigned the numbers 1 and 0 to describe the presence and absence, respectively, of organ involvement for the eye, external ear, inner ear, nasal cavity, respiratory system, cardiovascular system, renal system, skin, joints, and CNS.^[9]^ From the correlation matrix, we found a significant inverse relationship between the incidence of respiratory involvement and that of auricular involvement. We observed a positive relationship between respiratory involvement and nasal involvement. Auricular involvement was associated with cardiovascular involvement and renal involvement in RP patients. It is possible that organ involvement patterns with a focus on respiratory and auricular involvement may provide important diagnostic and prognostic information for RP patients.

We divided RP patients into 2 subgroups by the patterns of clinical manifestations, namely a subgroup of patients with respiratory involvement (R subgroup) and a subgroup of patients with auricular involvement (A subgroup), and investigated the clinical and laboratory characteristics of each subgroup.

## 2 Materials and methods

### 2.1 A multi-institutional study survey

We conducted an epidemiological survey using a questionnaire for collecting clinical information.^[1]^ The questionnaire assessed patient profiles (gender, onset age, and follow-up years), clinical features, laboratory findings (presence or absence of increased erythrocyte sedimentation rate, serum C-reactive protein, matrix metalloproteinase (MMP)3, and ferritin), imaging and pathological findings, treatment (medicines and surgical interventions), prognosis (patients taking no medications, well-controlled patients, limited responders, patients with progressive disease course, and death), and complications. A total of 395 physicians in Japan responded to the questions. Detailed clinical symptoms used in the questionnaire are summarized in Table 1. In this study, we compared patients’ profiles, clinical features, laboratory findings, medicines (corticosteroids, immunosuppressants, and biological agents), and prognosis.

**Table 1:**
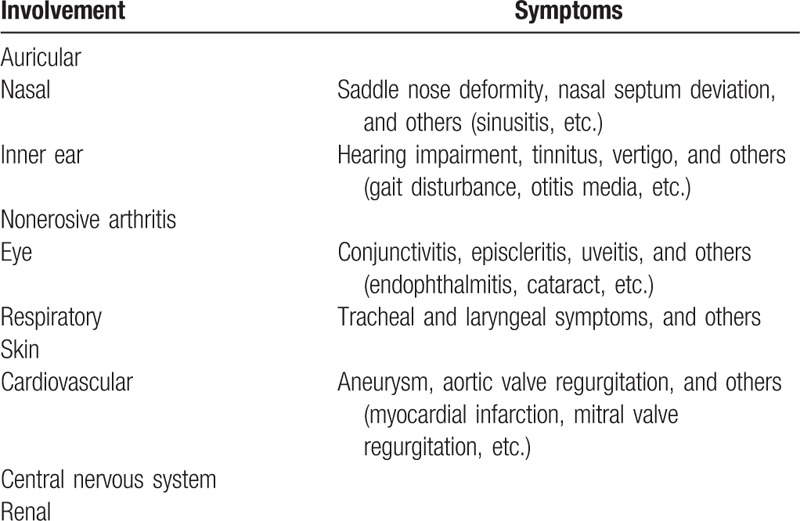
Clinical feature questionnaire.

### 2.2 Subgroup definition

According to our previous study,^[9]^ we categorized 239 RP patients into 2 subgroups by the patterns of clinical manifestations, namely a subgroup of patients with respiratory involvement without auricular involvement (termed the R subgroup) and patients with auricular involvement without respiratory involvement (termed the A subgroup). After dividing RP patients into 2 subgroups, we found a third subgroup of patients that had both auricular and respiratory involvement (termed the O subgroup for overlap) even though the patients within the O subgroup had a longer history of RP than the other subgroups. Four patients from a total of 239 patients in our initial survey^[2]^ were excluded because they did not fall into the R, A, or O subgroups.

### 2.3 ELISA for matrix metalloproteinase 3 (MMP3)

We obtained peripheral blood from 22 RP patients from a newly recruited patient cohort and from 11 normal individuals for ELISA assays. This study was approved by the institutional review boards of St. Marianna University School of Medicine and was registered with the University Hospital Medical Information Network-Clinical Trials Registry (UMIN000018937). We conducted this research according to the principles expressed in the Declaration of Helsinki. We obtained written informed consent from each individual prior to enrolment. A copy of the written consent is available for review upon request.

We assessed serum MMP3 concentrations using commercial ELISA kits (R&D Systems, Minneapolis, MN).

### 2.4 Data analysis

We compared the clinical characteristics using a Student's *t*-test with dummy variables in Tables 2 and 3. The presence and absence of clinical and laboratory findings were the dummy variables, 1 and 0, respectively.

**Table 2:**
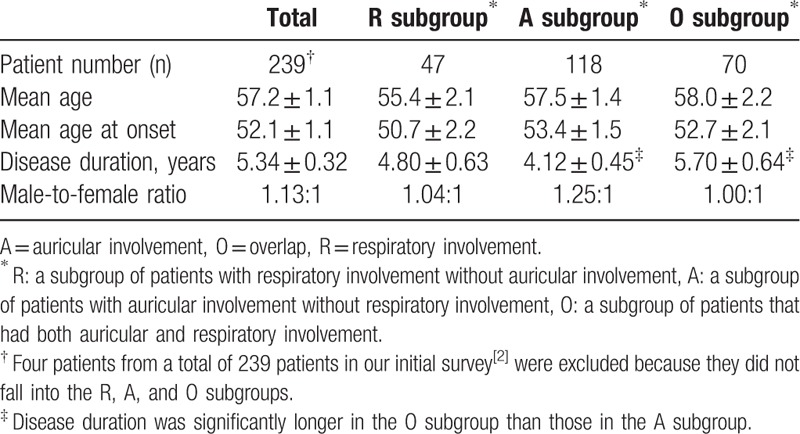
Demographic data of three subgroups in 239 Japanese RP patients.

**Table 3:**
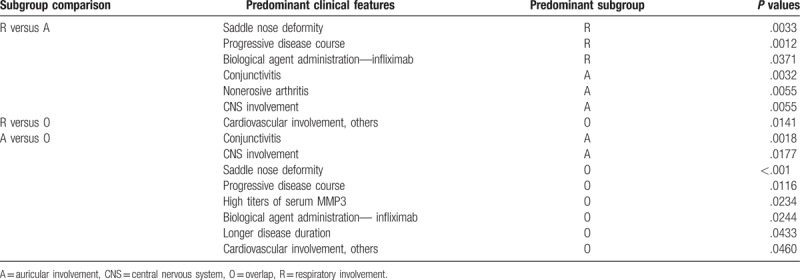
Predominant clinical characteristics demonstrated by the comparison between indicated subgroups.

Age and disease duration in Table 2 were expressed as mean ± standard error. We compared ELISA titers using a Wilcoxon rank sum test. Male-to-female ratios were compared by Fisher's exact test. A *P* value <.05 was considered significant.

We conducted an exploratory analysis using a correlation matrix among major RP complications with the dummy variables.^[9]^

We obtained correlation coefficients with *P* < .05 using the R package cor2.test. A correlation coefficient *r* = 0.13 (*P* = .045) was defined as the minimum significant *r*.

Each parameter was analyzed with statistical software JMP 13.0.0 (SAS Institute Japan, Tokyo, Japan).

## 3 Results

### 3.1 Demographical data of the patients

We divided 239 RP patients into the R subgroup or A subgroup. Patients in the R subgroup had respiratory involvement but not auricular involvement, and patients in the A subgroup had auricular involvement but not respiratory involvement. There were 47 (19.7%) and 118 (49.4%) patients in the R and A subgroups, respectively. We did not find significant differences in the age of disease onset, disease duration, and male-to-female ratio between the R and A subgroups. The remaining 70 patients (29.3%) constituted the third subgroup of RP patients with both respiratory and auricular involvement (termed the O subgroup for overlap). A significantly longer mean disease duration in the O subgroup (mean ± standard error, 5.70 ± 0.64 years) was observed than in the A subgroup (4.12 ± 0.45 years, Tables 2 and 3). The mean disease duration of the O subgroup was longer than that of the R subgroup (4.80 ± 0.63 years), but the difference was not significant.

### 3.2 Subgroup analyses of clinical characteristics in RP patients

#### 3.2.1 Comparison between the R and A subgroups

We compared the clinical characteristics of the R subgroup with those of the A subgroup (Table 3). In the questionnaire,^[2]^ physicians indicated patients’ prognosis from 5 items: patients taking no medications, well-controlled patients, limited responders, patients with a progressive disease course, and death. We found that saddle nose deformity and progressive disease course were observed frequently in the R subgroup.

Conjunctivitis, nonerosive arthritis, and CNS involvement were more prevalent in the A subgroup (Table 3) consistent with our previous study. Briefly, nasal involvement was significantly associated with respiratory involvement (*r* = 0.28),^[9]^ respiratory complications were the most common cause of death (55% of total deaths),^[2]^ eye involvement was significantly associated with joint involvement (*r* = 0.18),^[9]^ and auricular involvement was significantly associated with CNS involvement (*r* = 0.15).^[9]^

#### 3.2.2 Clinical characteristics of the O subgroup

Four patients were unable to be allocated to the R, A, or O subgroups. We compared the clinical characteristics of the O subgroup with those of the R subgroup (Table 3). Cardiovascular involvement, including myocardial infarction, angina, and mitral valve regurgitation, was predominant in the O subgroup (Table 3). Cardiovascular involvement was significantly associated with auricular involvement (r = 0.14), CNS involvement (*r* = 0.20), and renal involvement (*r* = 0.30).^[9]^ The mean disease duration of 17 patients with cardiovascular involvement (7.81 ± 2.2 years) was longer than that of 222 patients without involvement (5.40 ± 0.3 years, *P = *.06; not significant).^[5]^ In the 17 RP patients with cardiovascular involvement,^[2,5]^ 0 (0%), 9 (53%), and 8 (47%) patients were allocated to the R, A, and O subgroups, respectively. Patients in the R subgroup did not develop cardiovascular involvement, an important cause of death.^[5]^

A comparison between the O and A subgroups revealed the prevalence of saddle nose deformity, progressive disease course, high titers of MMP3, and cardiovascular involvement in the O subgroup. Conjunctivitis and CNS involvement were frequently observed in the A subgroup (Table 3).

Patients with inner ear involvement, renal involvement, and skin involvement were evenly allocated among the three subgroups. We did not find significant differences in the elevated erythrocyte sedimentation rate, serum C-reactive protein, and ferritin among the three subgroups.

### 3.3 Serum MMP3 concentrations

**Figure 1:**
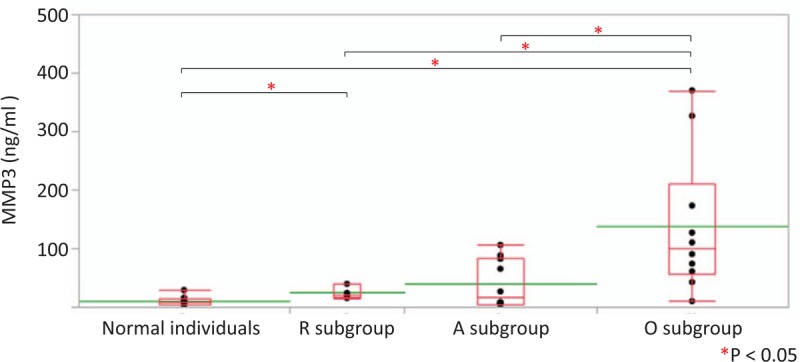
Serum MMP3 concentrations in the RP subgroups and normal individuals (normal individual subgroup). We obtained 26 sera from 22 RP patients and 11 sera from 11 normal individuals and measured MMP3 concentrations by ELISA. The 22 patients were divided into three subgroups: the R subgroup (5 patients, 23%), the A subgroup (9 patients, 41%), and the O subgroup (8 patients, 36%). We did not find any significant differences in age, age at onset, disease duration, and male-to-female ratio among the four subgroups including normal individuals. The R subgroup included 2 RP patients with nasal involvement according to the data from our previous study.^[9]^ We observed significantly higher MMP3 concentrations in the O subgroup than in the other subgroups including normal individuals. MMP3 concentrations in the R subgroup were significantly higher than in normal individuals. MMP3 concentrations are displayed with dot plots. A box-plot and a mean level (green line) for each subgroup are indicated. A = auricular involvement, MMP3 = matrix metalloproteinase-3, O = overlap, R = respiratory involvement, RP = relapsing polychondritis.

We obtained 26 sera from 22 newly recruited RP patients for the measurement of MMP3 concentrations. The 22 patients included 5 patients from the R subgroup (23%), 9 patients from the A subgroup (41%), and 8 patients from the O subgroup (36%). We compared the data with those of 11 normal individuals. Two RP patients with nasal involvement were allocated to the R subgroup.^[9]^ We observed significantly higher levels of MMP3 concentrations in the O subgroup than in the remaining subgroups including normal individuals (Fig. 1). MMP3 concentrations in the R subgroup were significantly higher than in normal individuals (Fig. 1).

## 4 Discussion

We found that patients with a progressive disease course were observed more frequently in the R subgroup than in the A subgroup. CNS involvement was observed more frequently in the A subgroup than in the R subgroup. The remaining RP patients formed the third O (overlap) subgroup and had both respiratory and auricular involvement. In the O subgroup, disease duration was significantly longer than that of the A subgroup (Tables 2 and 3). Cardiovascular involvement was observed predominantly in the O subgroup compared with the R and A subgroups. Furthermore, high titers of serum MMP3 concentrations were observed more frequently in the O subgroup than in the R and A subgroups.

Recently, Dion et al^[8]^ conducted a retrospective study of 142 RP patients in France and summarized patient characteristics. We compared the French data with our survey data.^[2]^ We noticed that the incidence of major RP complications was lower in Japan than in France. For example, lower incidences of auricular involvement (89% in France to 78% in Japan), joint involvement (69%–39%), eye involvement (56%–46%), nasal involvement (63%–39%), skin involvement (28% to 13%), valvular involvement (22%–2.1%), and MDS (8.5%–2.1%) were observed in patients with RP in Japan.^[2,5,7]^ In contrast, airway involvement (50% in France to 50% in Japan) and CNS involvement (7.7%–9.6%) were comparable in France and Japan and renal involvement (0% to 6.7%) was higher in Japan.^[2,7,10]^

We calculated the correlation coefficients of the incidence of major RP complications in 239 patients.^[9]^

We found a significant inverse relationship between respiratory involvement and external ear involvement (*r* = −0.48). In addition, we found a significant positive relationship between cardiovascular and renal involvement (*r* = 0.30), cardiovascular and CNS involvement (*r* = 0.20), cardiovascular and auricular involvement (*r* = 0.14), CNS and auricular involvement (*r* = 0.15), and respiratory and nasal involvement (*r* = 0.28).

Dion et al. found three clusters in their 142 RP patients and emphasized the importance of cardiac involvement and hematological disorders as poor prognostic factors.^[8]^ They found a significant inverse relationship between respiratory involvement and auricular involvement (*r* = −0.245),^[11]^ but they did not find a significant association between respiratory involvement and nasal involvement (*r* = 0.08). These results suggest that there exists similar patient subgroups in France^[11]^ and Japan, despite minor differences in demographic and clinical characteristics.

Although respiratory involvement was a major cause of death in France, Dion et al. reported that cardiovascular involvement was found most frequently in the most severe cluster (hematological cluster) and many patients did not develop respiratory involvement. We found a similar trend in Japanese patients with RP in that patients in the R subgroup did not develop cardiovascular involvement, which suggests that respiratory and cardiovascular involvement were mostly mutually exclusive both in France and in Japan.

Serum MMP3 concentrations were significantly higher in the O subgroup than in the R and A subgroups from another 22-patient cohort (Fig. 1). In a subgroup analysis of 239 patients, high MMP3 concentrations and longer disease duration were observed in the O subgroup compared with the A subgroup (Tables 2 and 3). Disease duration in the O subgroup was longer than that of the R subgroup, but the difference was not significant.

Chondrocytes in damaged cartilage strongly express MMP3 in RP.^[12]^ The number of MMP3-expressing chondrocytes correlated with apoptotic cell number in cartilage tissues.^[12]^ High MMP3 titer may suggest not only an active disease but also both auricular and respiratory involvement (overlapping involvement) in patients with RP.

In our original survey,^[2]^ we found that the incidence of auricular and respiratory involvement at onset was 57.3% and 17.2%, respectively. During a follow-up period (median 5.3 years), auricular and respiratory involvement occurred in 78.2% and 49.8% of RP patients, respectively. The degree of multiple organ involvement may be associated with gradual disease progression of RP patients with a longer disease history.

This study has the typical limitations of a retrospective study. We conducted a survey of physicians at major medical facilities in Japan, who have treated and/or are treating patients with RP to control for systemic biases.^[2]^ To simplify the survey process, we used qualitative variables to assess the laboratory findings. For example, in the questionnaire we asked about the presence or absence of high serum MMP3 titers in RP patients instead of working with the raw titer numbers. The O (overlapping) subgroup had higher MMP3 concentrations than the A (auricular) subgroup (Table 3).

Here, we conducted an ELISA assay of serum MMP3 concentrations and investigated the relationship of the MMP3 concentrations and organ involvement by recruiting 22 new RP patients. The sample size of the newly recruited patients was small, which may explain why we did not find any association between MMP3 concentrations and the clinical characteristics of the 22 newly recruited patients. It is still possible that MMP exerts an important role for disease progression. Thus, we will continue to recruit more RP patients to compare serum MMP3 concentrations and organ involvement patterns.

In conclusion, we divided RP patients into 2 subgroups, the R subgroup and A subgroup, based on respiratory and auricular involvement. Patients in the R subgroup did not develop cardiovascular involvement. CNS involvement, another major RP prognostic factor, was frequently observed in the A subgroup. The remaining patients formed a third O subgroup and had both respiratory and auricular involvement, which were both important prognostic factors in patients with RP. The current study may provide new insights into the classification and treatment of RP.

## Author contributions

**Conceptualization:** Jun Shimizu, Noboru Suzuki.

**Data curation:** Jun Shimizu, Yoshihisa Yamano, Noboru Suzuki.

**Formal analysis:** Jun Shimizu, Noboru Suzuki.

**Funding acquisition:** Noboru Suzuki.

**Investigation:** Jun Shimizu, Yoshihisa Yamano, Kimito Kawahata, Noboru Suzuki.

**Methodology:** Jun Shimizu, Yoshihisa Yamano, Kimito Kawahata, Noboru Suzuki.

**Project administration:** Jun Shimizu.

**Resources:** Jun Shimizu, Kimito Kawahata.

**Software:** Jun Shimizu.

**Supervision:** Yoshihisa Yamano, Kimito Kawahata, Noboru Suzuki.

**Validation:** Jun Shimizu, Noboru Suzuki.

**Visualization:** Jun Shimizu, Noboru Suzuki.

**Writing – original draft:** Jun Shimizu.

**Writing – review & editing:** Yoshihisa Yamano, Kimito Kawahata, Noboru Suzuki.

## References

[1] Letko E Zafirakis P Baltatzis S Relapsing polychondritis: a clinical review. Semin Arthritis Rheum 2002;31:384–95. 12077711 10.1053/sarh.2002.32586

[2] Oka H Yamano Y Shimizu J A large-scale survey of patients with relapsing polychondritis in Japan. Inflamm Regen 2014;34:149–56.

[3] Gergely P Jr Poór G Relapsing polychondritis. Best Pract Res Clin Rheumatol 2004;18:723–38. 15454129 10.1016/j.berh.2004.05.012

[4] Suzuki N Shimizu J Oka H Neurological involvement of relapsing polychondritis in Japan: an epidemiological study. Inflamm Regen 2014;34:206–8.

[5] Shimizu J Oka H Yamano Y Cardiac involvement in relapsing polychondritis in Japan. Rheumatology (Oxford) 2016;55:583–4. 26361883 10.1093/rheumatology/kev320

[6] Francès C el Rassi R Laporte JL Dermatologic manifestations of relapsing polychondritis. A study of 200 cases at a single center. Medicine (Baltimore) 2001;80:173–9. 11388093 10.1097/00005792-200105000-00003

[7] Shimizu J Oka H Yamano Y Cutaneous manifestations of patients with relapsing polychondritis: an association with extracutaneous complications. Clin Rheumatol 2016;35:781–3. 26780448 10.1007/s10067-015-3160-2

[8] Dion J Costedoat-Chalumeau N Sène D Relapsing polychondritis can be characterized by three different clinical phenotypes: analysis of a recent series of 142 patients. Arthritis Rheumatol 2016;68:2992–3001. 27331771 10.1002/art.39790

[9] Shimizu J Yamano Y Yudoh K Organ involvement pattern suggests subgroups within relapsing polychondritis: comment on the article by Dion et al. Arthritis Rheumatol 2018;70:148–9. 28941194 10.1002/art.40330

[10] Shinomiya F Mima N Nanba K Life expectancies of Japanese patients with rheumatoid arthritis: a review of deaths over a 20-year period. Mod Rheumatol 2008;18:165–9. 18317879 10.1007/s10165-008-0031-6

[11] Dion J Costedoat-Chalumeau N Piette JC Reply to Shimizu et al. “Organ involvement pattern suggests subgroups within relapsing polychondritis”. Arthritis Rheumatol 2018;70:149. 10.1002/art.40330 28941194

[12] Ouchi N Uzuki M Kamataki A Cartilage destruction is partly induced by the internal proteolytic enzymes and apoptotic phenomenon of chondrocytes in relapsing polychondritis. J Rheumatol 2011;38:730–7. 21239745 10.3899/jrheum.101044

